# Genome Maintenance Mechanisms at the Chromatin Level

**DOI:** 10.3390/ijms221910384

**Published:** 2021-09-27

**Authors:** Hirotomo Takatsuka, Atsushi Shibata, Masaaki Umeda

**Affiliations:** 1 School of Biological Science and Technology, College of Science and Engineering, Kanazawa University, Kakuma-Machi, Kanazawa 920-1192, Japan; h-takatsuka@se.kanazawa-u.ac.jp; 2 Signal Transduction Program, Gunma University Initiative for Advanced Research (GIAR), 3-39-22, Showa-Machi, Maebashi 371-8511, Japan; shibata.at@gunma-u.ac.jp; 3 Graduate School of Science and Technology, Nara Institute of Science and Technology, Ikoma 630-0192, Japan

**Keywords:** DNA damage, DNA double-strand break, DNA repair, genome integrity, chromatin, epigenetics, histone methylation, histone acetylation

## Abstract

Genome integrity is constantly threatened by internal and external stressors, in both animals and plants. As plants are sessile, a variety of environment stressors can damage their DNA. In the nucleus, DNA twines around histone proteins to form the higher-order structure “chromatin”. Unraveling how chromatin transforms on sensing genotoxic stress is, thus, key to understanding plant strategies to cope with fluctuating environments. In recent years, accumulating evidence in plant research has suggested that chromatin plays a crucial role in protecting DNA from genotoxic stress in three ways: (1) changes in chromatin modifications around damaged sites enhance DNA repair by providing a scaffold and/or easy access to DNA repair machinery; (2) DNA damage triggers genome-wide alterations in chromatin modifications, globally modulating gene expression required for DNA damage response, such as stem cell death, cell-cycle arrest, and an early onset of endoreplication; and (3) condensed chromatin functions as a physical barrier against genotoxic stressors to protect DNA. In this review, we highlight the chromatin-level control of genome stability and compare the regulatory systems in plants and animals to find out unique mechanisms maintaining genome integrity under genotoxic stress.

## 1. Introduction

Plants are sessile organisms that are constantly threatened by a variety of stressors that damage their genomic DNA. To cope with such life-threatening challenges, plants have evolved a distinct system of DNA damage response (DDR), which triggers a cell-cycle checkpoint and enables DNA repair. DNA repair machinery that has been studied extensively in other eukaryotes, such as yeasts and mammals, are highly conserved in plants [1]. For instance, pyrimidine dimers produced on ultraviolet (UV)-irradiated DNA are repaired by photoreactivation [2]. Two types of excision repair mechanisms, base excision repair and nucleotide excision repair, have been shown to repair various types of DNA lesions [3]. Incorrectly paired nucleotides and UV-induced photolesions are removed via mismatch repair [4,5]. DNA single- and double-strand breaks (SSBs and DSBs, respectively) are resolved through homologous recombination (HR) and nonhomologous end joining (NHEJ) [6,7]. Nevertheless, plants also possess unique cell-cycle checkpoint mechanisms. In animals, ATAXIA-TELANGIECTASIA MUTATED (ATM) kinase is activated on sensing DSBs to arrest the cell cycle at the G1/S or G2/M phase [8,9]. ATAXIA-TELANGIECTASIA-AND-RAD3-RELATED (ATR) kinase acts as another checkpoint kinase at G2/M or intra-S after the recognition of single-stranded DNA (ssDNA) and stalled replication forks [10,11]. Plants also have functional ATM and ATR, whereas downstream regulators orthologous to mammalian counterparts are all missing. Instead, the plant-specific transcription factor SUPPRESSOR OF GAMMA RADIATION 1 (SOG1), which is phosphorylated and activated by ATM and ATR, has been shown to play an essential role in DDR [12,13]. Recent studies have uncovered SOG1-dependent pathways causing G2 arrest in response to DSBs in *Arabidopsis thaliana* [14,15,16,17,18].

In response to DSBs, plants induce G2 arrest in transit-amplifying cells, but also promote an early onset of endoreplication, which repeats DNA replication without mitosis or cytokinesis, thereby enhancing cell growth and differentiation [16,18,19,20,21,22]. To prevent mutated cells from dividing and ensure organ growth by increasing cell volume, plants have deployed an active mechanism to evoke endoreplication in response to DSBs. In mammals, severe DNA damage generally causes cell death, whereas DSBs specifically trigger stem cell death in plant meristems through the ATM-SOG1 pathway [19,23,24]. As plant cells cannot migrate within tissues, cell death usually injures tissue structure and inhibits organ growth. Therefore, stem-cell-specific death is probably beneficial for the continuous development of plants, while decreasing the risk of descendant cells inheriting incorrect genetic information.

The risk of DNA being exposed to external or internal genotoxic stresses and the feasibility of DNA repair highly depend on whether DNA is exposed at the surface of chromatin or is buried deep inside the chromatin jungle, which is composed of four core histones (H2A, H2B, H3, and H4) and one linker histone (H1) [25]. The chromatin structure is dynamically and reversibly reorganized in response to developmental and environmental cues. The conversion of the chromatin structure from an open euchromatic state to a closed heterochromatic state, and vice versa, is regulated by two types of chromatin modifications: DNA methylation and histone modifications. DNA methylation is a process by which methyl groups are attached to DNA in a reversible manner [26]. DNA methylation-specific binding proteins are known to locally alter the state of histone modifications, thereby affecting the chromatin structure [26]. Histone modifications are classified into at least eight types: acetylation, methylation, phosphorylation, ubiquitylation, GlcNAcylation, citrullination, krotonilation, and isomerization [27,28]. The chromatin structure is controlled by histone modifications through at least two mechanisms: (1) the net charge of histones is altered by histone modifications, thereby changing their DNA-binding activity, and (2) modified histones serve as docking sites for proteins determining the chromatin architecture [27,28]. In plants, the acetylation and methylation of H3 and H4 have been well characterized. For example, typical euchromatic modifications comprise the acetylation of histone H3 lysine 9 (H3K9ac) and histone H4 lysine 5 or 14 (H4K5ac or H4K14ac); mono-, di-, or tri-methylation of histone H3 lysine 4 (H3K4me1, H3K4me2, or H3K4me3); and di- or tri-methylation of histone H3 lysine 36 (H3K36me2 or H3K36me3) [29,30]. The di-methylation of histone H3 lysine 9 (H3K9me2) and mono- or tri-methylation of histone H3 lysine 27 (H3K27me1 or H3K27me3) also play crucial roles in heterochromatin formation [29,30].

This review summarizes the current understanding of plant strategies to cope with DNA damage at the chromatin level, focusing on three facets: (1) the control of chromatin modifications at damaged sites for efficient DNA repair, (2) the epigenetic regulation of gene expression required for DDR, and (3) possible roles of the chromatin structure as a physical barrier to protect DNA from genotoxic stressors. For the first two topics, the basic information has been covered in other review articles (see [31,32]); in this review, we will mainly focus on the latest findings that will be important milestones for this field in the future. The last topic sheds light on a concept that has recently been gaining attention in plants. Since this idea is still in the beginning stages of being established, we will summarize the findings obtained thus far and discuss issues that need to be resolved in the future. Compared to animals, the information available on plants is limited; therefore, in each section, we first introduce basic knowledge in animals and then provide recent findings in the plant field. We do not cover chromatin remodelers or histone chaperones, instead illuminating the chromatin modifications that regulate DDR.

## 2. Control of Chromatin Modifications at Damaged Sites

Chromatin modifications around damaged sites have a significant impact on DNA repair; namely, specific chromatin modifications can function as a scaffold for DNA repair machineries. It is also known that alterations in particular chromatin modifications decrease chromatin compaction, thereby enabling DNA repair proteins to have easy access to the damaged sites.

### 2.1. In Animals

**Figure 1:**
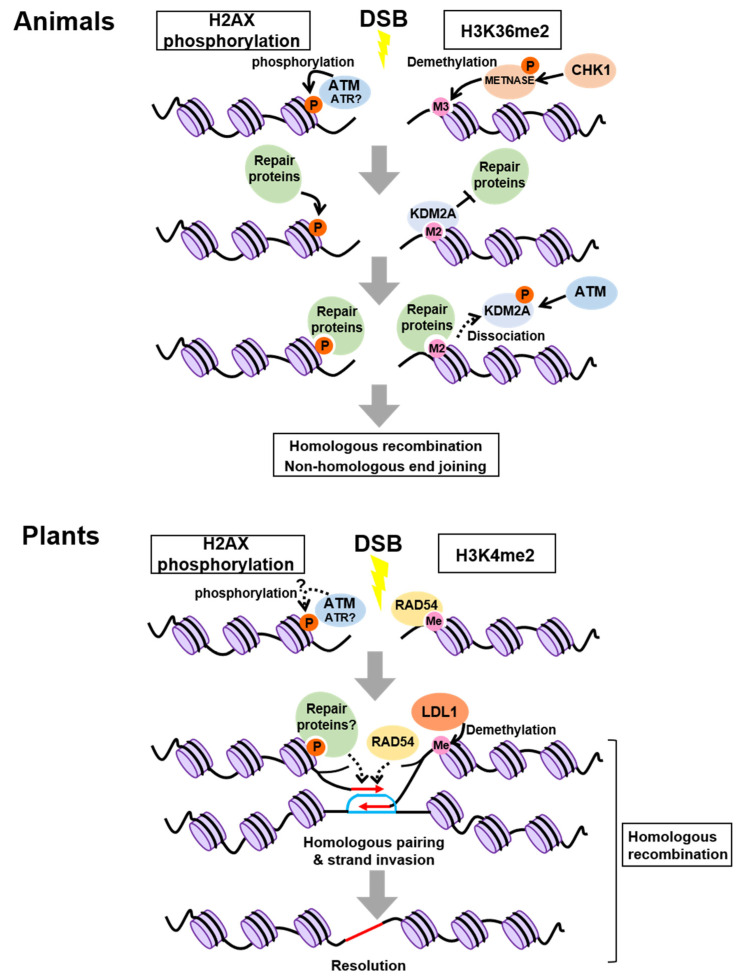
Chromatin modifications involved in homologous recombination and nonhomologous end joining. In animals, ATM is known to phosphorylate H2AX, whereas the involvement of ATR has not been reported so far. In plants, it remains unknown whether H2AX is phosphorylated by ATM or ATR, and whether H3K4 di-methylation is enhanced specifically around damaged sites. M2 and M3 represent H3K36 di- and tri-methylation, respectively. Red and blue lines represent RAD51-bound ssDNA and the captured homologous sequence, respectively.

The best-studied histone modification in DDR is phosphorylation of the histone H2A variant H2AX; hereafter, phosphorylated H2AX is referred to as γH2AX. H2AX is phosphorylated at the sites flanking DSBs in an ATM- or ATR-dependent manner, thereby attracting DNA repair proteins [33,34,35] (Figure 1). While γH2AX is assumed to be a DSB-specific histone modification, recent studies have revealed that other histone modifications, which are already established prior to DNA damage, also change at the damaged sites. For instance, the SET domain protein METNASE, which is phosphorylated by CHECKPOINT KINASE 1 (CHK1), a protein kinase acting downstream of ATR, promotes the conversion of tri-methylation to di-methylation of H3K36 around damaged sites after DSB induction [36,37] (Figure 1). The newly formed H3K36me2 is initially masked through physical interaction with LYSINE DEMETHYLASE 2A (KDM2A), a specific demethylase of H3K36me2, but ATM-dependent phosphorylation of KDM2A causes its dissociation from H3K36me2 and thereby exposes the histone mark to several proteins required for NHEJ and HR [38] (Figure 1). Similarly, H3K4me3, H3K9me3, H3K27me3, H4K20me2, and H4ac function in DNA repair, although their recruited proteins differ from those of H3K36me2 [39].

Constitutive heterochromatin is tightly packed and, thus, forms a challenging environment for DNA repair. To repair heterochromatic DSBs, a multistep reaction involving chromatin remodeling is indispensable. Following the induction of DSBs by ionizing radiation, ATM phosphorylates KRAB-ASSOCIATED PROTEIN 1 (KAP1), which induces heterochromatin relaxation, leading to the release of CHROMODOMAIN HELICASE DNA-BINDING PROTEIN 3 (CHD3) from chromatin [40,41,42,43]. Specifically, in the G2 phase, the ATM-KAP1-dependent chromatin remodeling promotes 5′-end resection at a heterochromatic DSB to generate ssDNA [40,41,43]. Following the remodeling and resection, RAD51, which forms a nucleoprotein filament with ssDNA, promotes the DNA strand exchange reaction and captures double-stranded DNA to find a homologous sequence [44], thereby enhancing the rate of homologous recombination (HR) [44]. Thus, the ATM-KAP1-dependent chromatin remodeling is a critical step in efficiently repairing heterochromatic DSBs [40,41,42] (see further details in [45]).

Heterochromatin remodeling in response to DSBs also requires the alteration in chromatin modifications; H3K9me2/me3 and H3K56me2/me3 are subjected to demethylation at heterochromatic DSBs, but not at euchromatic DSBs in *Drosophila* cells [46]. This results in increased H3K9me1 and H3K56me1, as well as decompaction of damaged sites, thus enhancing the accessibility of DNA repair proteins [46].

### 2.2. In Plants

As in animals, γH2AX has been well characterized in plants. Immunostaining experiments with the antibody against *Arabidopsis* γH2AX showed that the number of γH2AX foci increased after ionizing radiation in a dose-dependent manner; therefore, γH2AX foci are good indicators of DNA damage in plant cells [47]. *Arabidopsis* possesses two genes encoding the H2AX isoforms, H2AXA and H2AXB, differing in amino acid sequences only at two residues. However, the role of their phosphorylation in DDR has been a long-standing question. Waterworth et al. [48] recently demonstrated that *h2axa/b* double mutants were hypersensitive to mitomycin C, an inducer of DNA damage via DNA alkylation that generates interstrand cross-links. In this study, the wild type but not the non-phosphorylatable form of H2AX could complement the mutant phenotype, suggesting an essential role of H2AX phosphorylation in plant DDR. DNA damage-dependent phosphorylation of H2AX is abolished in the *atm atr* double mutant of *Arabidopsis*, indicating its requirement for ATM and ATR, as reported in animals (Figure 1) [49]. However, there is no clear evidence that ATM or ATR directly phosphorylates H2AX in plant cells.

A recent study demonstrated that H3K4me2, representing one of the histone marks involved in transcriptional activation, plays an important role in recruiting a key regulator for HR in *Arabidopsis* [50]. RAD54, a member of the switch/sucrose non-fermentable (SWI2/SNF2) family, interacts with the RAD51-ssDNA filament to stabilize its structure, thereby elevating the rate of HR [51] (Figure 1). Hirakawa et al. [50] revealed that RAD54 recognizes and binds directly to H3K4me2 at the damaged sites, implying that H3K4me2 acts as a hallmark for RAD54-mediated HR repair (Figure 1). Moreover, co-immunoprecipitation using γ-irradiated *Arabidopsis* seedlings carrying YFP-tagged RAD54 identified the H3K4me2 demethylase LYSINE-SPECIFIC DEMETHYLASE1-LIKE 1 (LDL1). Interestingly, depletion of LDL1 led to overaccumulation of RAD54 at damaged sites and delayed HR, suggesting that to perform efficient HR, dissociation of RAD54 from the nucleoprotein filament needs to occur at the right time through LDL1-mediated demethylation of H3K4me2 [50] (Figure 1). To our knowledge, a direct interaction between RAD54 and histone methylation has not been described thus far in animals; therefore, plants might have developed a distinct system for HR by establishing the unique RAD54-H3K4me2 link. However, it remains elusive whether the H3K4me2 status is indeed altered around the damaged sites. Developing technologies for the live-imaging of H3K4me2 will help understand the initial event in HR.

The mono-methylation of H3K27, which is catalyzed by the plant-specific histone methyltransferases ARABIDOPSIS TRITHORAXRELATED 5 (ATXR5) and ATXR6, is involved in the formation of constitutive heterochromatin [52]. It has been reported that a reduced H3K27me1 level in the *atxr5/6* double mutant causes the overreplication of heterochromatic regions and results in the accumulation of heterochromatic DNA breaks, even without treatment with exogenous genotoxic agents [53,54]. Intriguingly, overreplication-associated DNA damage induces a dynamic remodeling of centromeric heterochromatin into the unique structure, named an “overreplication-associated center (RAC),” which is composed of three ring-shaped layers, each with distinct components: (1) an outer layer of condensed heterochromatin represented by 4′,6-diamidino-2-phenylindole (DAPI)-dense foci, (2) a H2AX-rich inner layer, and (3) a low-density core containing foci of γH2AX and RAD51 [54]. DAPI-dense foci are missing in RACs except for the outermost layer, suggesting that the interior region of RACs is less compacted to provide a site for efficient DNA repair. However, the interior region is not enriched for typical euchromatic histone marks, implying that RACs are unique structures, harboring in their inner areas relaxed chromatin without acquiring a euchromatic state [54]. However, in wild-type plants, neither γ-irradiation nor hydroxyurea was sufficient to induce a higher density of heterochromatic breaks required for the formation of RAC-like structures [54]. Therefore, it remains controversial whether RACs are a bona fide plant-specific structure that facilitates DNA repair in heterochromatic regions, or an aberrant structure formed as a consequence of overreplication in the *atxr5/6* mutant.

Here, we have summarized the findings using a widely used model plant, *Arabidopsis thaliana*. However, importantly, recent studies have uncovered that DNA repair genes are conserved in other plant species, such as *Oryza sativa, Zea mays*, and *Saccharum officinarum* [55,56,57], suggesting that DNA repair machinery similar to those described above are also preserved across the plant kingdom.

## 3. Epigenetic Regulation of DDR-Related Genes

DNA damage is known to trigger genome-wide epigenetic reprogramming through histone methylation and acetylation, which alters the gene expression involved in DDR. In plants, such changes in gene expression are required for various cellular events, including cell death, cell-cycle arrest, and an early onset of endoreplication.

### 3.1. In Animals

**Figure 2:**
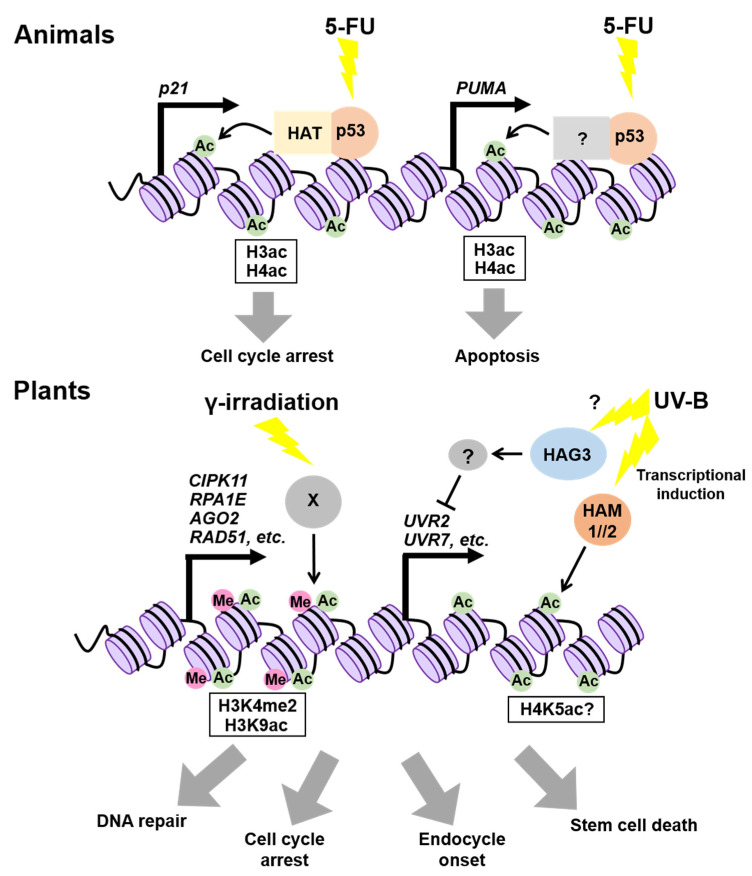
Epigenetic regulation of gene expression in response to genotoxic stress. In animal treated with 5-FU, p53 enhances the acetylation of histone H3 and H4 at the *p21* and *PUMA* loci, thereby inducing cell-cycle arrest and apoptosis. In plants, H3K9 acetylation and H3K4 di-methylation become enriched at the promoters of DDR genes upon γ-irradiation. Ultraviolet (UV)-B upregulates *HAM1* and *HAM2*, thereby elevating the expression of UV-B-induced genes (e.g., *UVR2* and *UVR7*) probably by increasing the H4K5ac level.

p53 is a transcriptional factor that prevents cancer by promoting DNA repair, cell-cycle arrest, and cell death in response to a variety of stressors, including genotoxic stress [58,59,60,61]. Previous studies have demonstrated that p53 exerts its function by controlling the histone acetylation on target gene promoters. The most well-known target of p53 is the gene for cyclin-dependent kinase (CDK) inhibitor p21, which binds to CDK and arrests the cell cycle [62] (Figure 2). In the presence of the anticancer drug 5-fluorouracil (5-FU), p53 binding to the *p21* promoter enhances the acetylation of both H3 and H4 by increasing the DNA-binding ability of two subunits of the histone acetyltransferase (HAT) complex, p300/CBP histone acetyltransferase and Transformation/Transcription Domain Associated Protein (TRRAP), in human cultured cells [62] (Figure 2). On 5-FU treatment, the acetylation levels of H3 and H4 on the promoter of *p53 UPREGULATED MODULATOR OF APOPTOSIS* (*PUMA*), which encodes a member of the B-CELL CLL/LYMPHOMA 2 (BCL-2) protein family, were also found to be elevated, thereby inducing its expression and consequently promoting apoptosis in human cells [63] (Figure 2). Thereafter, the genome-wide analysis of the p53-binding sites revealed that no prominent enrichment of H3K4me2 or H3K4me3 (14% or 3%, respectively) was identified, while acetylated histone H3 (H3K9/14ac) and H4 (H4K5/8/12/16ac) occupied 38% and 89% of the p53-binding sites, respectively [64]. Considering that frequencies of appearance of H3/H4ac and H3K4me2/3 were comparable in randomly selected genes, it is likely that p53-binding sites are highly enriched for histone acetylation [64]. Taken together, p53 seems to induce target genes by increasing histone acetylation on their promoters.

### 3.2. In Plants

SOG1, a member of the NAC [NO APICAL MERISTEM (NAM), ARABIDOPSIS TRANSCRIPTION ACTIVATION FACTOR (ATAF), and CUP-SHAPED COTYLEDON (CUC)] transcription factors, plays a crucial role in transmitting DNA damage signals in plants [12,13,65,66]. Previous studies demonstrated that SOG1 is required for DNA repair, cell-cycle arrest, an early onset of endoreplication, and stem cell death in response to DSBs [12,16,20,67,68]. To perform these cellular processes, SOG1 directly regulates more than 100 genes in *Arabidopsis*, e.g., *SIAMESE-RELATED 5* (*SMR5*) and *SMR7* for cell-cycle arrest, as well as *RAD51* and *RAD54* for DNA repair [17,69].

Both SOG1 and p53 are phosphorylated by ATM and ATR, and are involved in DDR, suggesting their functional similarity [65]. Although it remains unknown whether SOG1 regulates target genes by influencing chromatin modifications on their promoters, a previous study showed that the epigenetic status of some SOG1-direct target genes is altered after γ-irradiation [70]. In particular, the H3K4me2 levels at the loci of *CBL-INTERACTING PROTEIN KINASE 11* (*CIPK11*), *REPLICATION PROTEIN A 1E* (*RPA1E*), *ARGONAUTE 2* (*AGO2*), and *RAD51*, all of which are direct targets of SOG1, were elevated significantly after γ-irradiation in *Arabidopsis* (Figure 2) [70]. Another histone mark, H3K9ac, was also enriched on the *AGO2* promoter in response to γ-irradiation [70]. As many plant transcription factors are known to interact directly with chromatin modifiers to regulate the expression of target genes [71], it is likely that SOG1 also recruits chromatin modifiers of target gene promoters to elevate the level of active histone marks.

The *Arabidopsis* genome encodes 12 HATs and 18 histone deacetylases (HDACs), the former being classified into four types based on amino acid sequence similarities: GNAT (Gcn5-related N-acetyltransferase), p300/CBP, TAFII250, and MYST (MOZ, YbF2, Sas2, Tip60-like) families [72]. Among them, two MYST family members, HISTONE ACETYLTRANSFERASE OF THE MYST FAMILY 1 (HAM1) and HAM2, and one GNAT family member, HISTONE ACETYLTRANSFERASE OF THE GNAT FAMILY 3 (HAG3), participate in the response to UV-B that causes the formation of cyclobutane pyrimidine dimers (CPDs) [73]. Campi et al. [74] reported that *HAM1* and *HAM2* are upregulated upon UV-B irradiation, and that the UV-B-dependent induction of DNA repair genes, such as *UV RESISTANCE 2* (*UVR2*) and *UVR7*, is compromised in the *ham1* or *ham2* mutants, compared to wild types, leading to the overaccumulation of CPDs. HAM1 and HAM2 are known to function in globally maintaining H4K5ac [75]; therefore, they may also locally increase the H4K5ac level to upregulate *UVR2/7* in UV-B irradiation (Figure 2). However, it is also probable that HAM1 and HAM2 enhance other types of histone acetylation in response to UV-B. On the other hand, transcripts of UV-B-regulated genes, such as *UVR2* and *UVR7*, accumulate in the *hag3* knock-down lines even in the absence of UV-B irradiation [76], suggesting that HAG3 is involved in the repression of UV-B-regulated genes, presumably through the induction of unknown negative factor(s) (Figure 2). These observations, showing the opposite roles of HAM1/2 and HAG3 in UV-B response, together suggest distinct roles of plant HATs in fine-tuning DDR.

## 4. Possible Roles of Chromatin Structure as a Physical Barrier to Genotoxic Stress

As described above, a decrease in chromatin compaction facilitates the access of DNA repair proteins to damaged DNA. However, recent studies have indicated that the conversion of chromatin to a highly assembled structure contributes to the maintenance of genome integrity by providing a physical barrier against genotoxic stressors.

### 4.1. In Animals

Spermidine influences the folding of DNA molecules into a compact state in vitro. Based on this property, Yoshikawa et al. [77] developed a system to artificially manipulate the conformation of giant DNA molecules larger than 100 kbp. They reported that the frequency of γ-irradiation-induced DSBs in the presence versus absence of spermidine was approximately 4%, indicating that the compaction of DNA molecules greatly decreases the sensitivity to γ-irradiation. At the chromatin level, Mg^2+^ is known to have a positive effect on chromatin condensation. Thus, Takata et al. tested the Mg^2+^ treatment of isolated nuclei from HeLa cells attached on a glass slide to provoke a change in chromatin condensation [78]. Intriguingly, Mg^2+^-treated nuclei with condensed chromatin suffered from less DSBs after γ-irradiation compared to the control without Mg^2+^, suggesting that chromatin compaction can be a physical barrier to genotoxic stressors [78]. Given that γ-irradiation produces reactive radicals through the radiolysis of water molecules caught by chromatin, it is possible that condensed chromatin harbors fewer water molecules, thus reducing the likelihood of being exposed to reactive radicals [78]. However, a possibility still remains that chromatin condensation protects the genome from genotoxic agents that act directly on DNA molecules.

Findings from a recent study using mouse mesothelioma cells support the idea that chromatin condensation is involved in shielding DNA from damage. Brambilla et al. [79] investigated chromatin openness using two sequence-based methods: (1) transposase-accessible chromatin with high-throughput sequencing (ATAC-seq), identifying nucleosome-free regions, and (2) breaks labelling in situ and sequencing (BLISS), labeling DSB ends with double-stranded oligonucleotide adaptors [80,81]. Their data showed that γ-irradiation-induced DSBs accumulated in nucleosome-free regions of the genome [79].

It should be noted, however, that the conventional view that heterochromatin is just a physically packed, membraneless structure is not sufficient to account for the shielding role of heterochromatin, since water and oxygen molecules that are sources of reactive radicals would continuously flow from outside the heterochromatic domains. Nevertheless, importantly, heterochromatic domains were shown to form via liquid–liquid phase separation, which is a phenomenon giving rise to non-membrane-bound cellular compartments [82,83]. Furthermore, liquid droplets undergoing phase separation exhibited a limited permeability of exogenous molecules, shedding light on a critical role of phase separation in determining the composition and/or concentrations of substances inside the compartment [84]. Indeed, Strom et al. showed that small molecules, such as fluorescent dextrans, are excluded from phase-separated heterochromatic domains [83]. To further understand the functional role of chromatin as a physical barrier, it will be important to examine whether phase separation has an impact on the permeability of molecules that can damage DNA (e.g., water and oxygen) into heterochromatin.

### 4.2. In Plants

**Figure 3:**
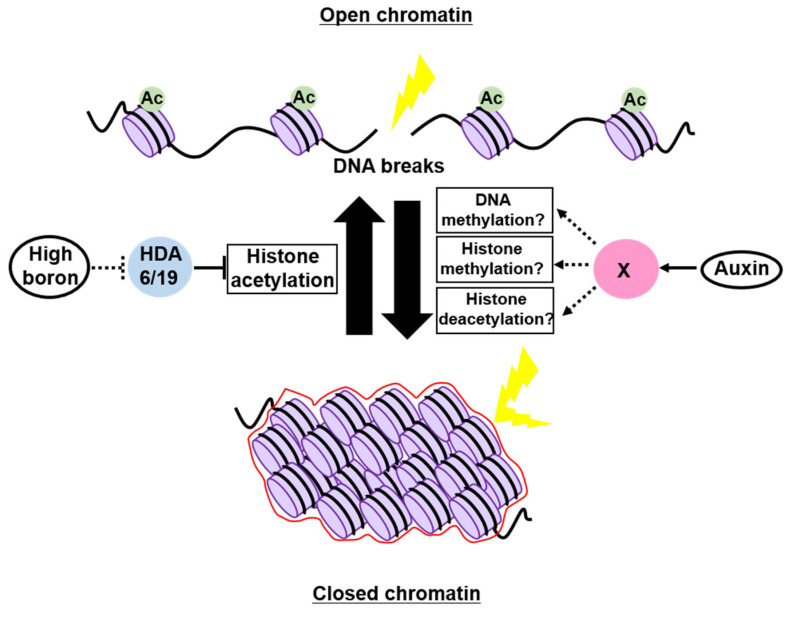
Epigenetic regulation of the chromatin structure involved in genome maintenance in plants. Excess boron opens up chromatin by enhancing histone acetylation, which may require the inhibition of HDACs, HDA6, and HDA19, thereby increasing the susceptibility to DNA damage. On the other hand, auxin promotes chromatin condensation and provides tolerance to genotoxic stress. The chromatin modifier(s) that function downstream of auxin signaling remain unknown.

Boron (B) is an essential micronutrient for plant growth and development [85], but a previous study demonstrated that excess B is the primary cause of DSBs in soil [86], but it remains poorly understood how high-B stress causes DNA breaks. Sakamoto et al. [87] discovered that the acetylation level of histone H3 was globally increased in *Arabidopsis* grown under high-B conditions, whereas the heterochromatic mark H3K9me1 was decreased, suggesting that high B impacts on relaxing chromatin by altering histone modifications. This study also showed that the occurrence of DSBs was positively correlated with the H3K9/14ac level in *Arabidopsis* roots, implying that high B opens chromatin at least through histone hyperacetylation, thereby elevating the susceptibility to genotoxic factors (Figure 3). Indeed, the mutants of *HISTONE DEACETYLASE 6* (*HDA6*) and *HDA19*, in which histone acetylation is increased at the genome-wide level, exhibited a reduced sensitivity to high-B stress [87] (Figure 3). However, HDA6 and HDA19 are known to interact with transcription factors to carry out locus-specific histone deacetylation [88,89,90]; therefore, their mutations might open specific loci encoding DNA repair-related genes, leading to lower susceptibility to high B.

A possible role of the phytohormone auxin in controlling the chromatin structure was described in suspension cultured cells. Hasegawa et al. [91] examined the chromatin accessibility in tobacco BY-2 cells using micrococcal nuclease (MNase), which preferentially digests DNA in regions where proteins are not stably bound [92]. When cells were cultured in the presence of a large amount of auxin, the genomic DNA became tolerant to MNase, indicating reduced chromatin accessibility. Conversely, when *Arabidopsis* cultured cells treated with PEO-IAA, an auxin antagonist blocking auxin signaling, were subjected to ATAC-seq analysis, nucleosome-free regions were found to be increased in the genome, especially in the gene body [91]. Interestingly, DSB accumulation after treatment with the DSB inducer zeocin was also alleviated by the simultaneous application of auxin, suggesting the role of auxin in maintaining genome integrity through chromatin condensation (Figure 3) [91]. This view is supported by transcriptomic data showing that, in addition to chromatin remodelers and histone chaperones, several chromatin modifiers involved in heterochromatin formation were repressed by PEO-IAA treatment [91]. However, it is noteworthy that plant cultured cells have experienced a variety of chromosomal rearrangements, such as translocation, deletion, and duplication, and display an aberrant epigenetic landscape compared to proliferating cells in tissues [93,94], making it difficult to conclude that auxin is generally involved in controlling the chromatin structure and susceptibility to genotoxic stress. Moreover, as auxin is essential for driving the cell cycle, during which the epigenetic status varies greatly [95,96,97], exogenous auxin or PEO-IAA application might have altered cell-cycle progression, consequently affecting the extent of chromatin condensation. Therefore, further careful experiments are definitely needed to assess the requirement of auxin in regulating the chromatin structure and genome stability.

Recently, Takahashi et al. [98] reported that in the *Arabidopsis* root tip, DSBs elevate the cytokinin level and repress the expression of some *PIN-FORMED* genes encoding auxin efflux carriers, thereby inhibiting downward auxin flow. The resultant reduction of auxin signaling causes cell-cycle arrest in the meristem and stem cell death, which contribute to maintaining genome integrity by providing time for DNA repair and removing DNA-damaged stem cells, respectively [98]. These data are consistent with a previous report showing that chilling stress-triggered DSBs induced columella stem cell death, which was suppressed by exogenous auxin treatment [99]. In the absence of genotoxic or chilling stress, auxin highly accumulates in the quiescent center (QC) and stem cells in the root tip of *Arabidopsis* [100,101]. These findings indicate that under stressful conditions, plants probably sacrifice the auxin-mediated control of genome integrity to prioritize cell-cycle checkpoints and stem cell renewal, which occurs after killing stem cells through activating cell division in the QC where DNA repair machinery is highly expressed to maintain genome integrity [102].

## 5. Concluding Remarks and Perspectives

Plants are much more tolerant to genotoxic stress than animals. For instance, 10-Gy irradiation reduces the survival rate of rats to about 20% of those in non-irradiated conditions [103], whereas *Arabidopsis* seedlings can survive 150-Gy γ-irradiation without showing any obvious defects in leaf production [12]. As plants are sessile organisms exposed to various external stresses causing DNA damage, it is conceivable that they might deploy a distinct regulatory system to protect their genomes from DNA damage. Considering that exogenously applied auxin, which has a promotive effect on chromatin condensation, further strengthens DNA damage tolerance in tobacco and *Arabidopsis*, as described above, chromatin organization might be one of the crucial factors that determine DNA damage tolerance in plants. Interestingly, a recent study reported that genome size is associated with the sensitivity to DNA damage in plants; plants with a larger genome size are more sensitive to DNA damage than those with smaller genomes [104]. This finding is counterintuitive because larger genomes are usually abundant with transposable elements that are major constituents of heterochromatin. Therefore, it is unlikely that the ratio of heterochromatin by itself can account for DNA damage tolerance. Furthermore, recent studies demonstrated that chromatin of animals and large-genome plants shapes topologically associating domains (TADs) and TAD-like structures, self-interacting genomic regions where DNA sequences physically interact with each other frequently [105,106,107]. Contrarily, *Arabidopsis,* which has a smaller genome, does not organize its chromatin into apparent TADs, suggesting that chromosomes can be partitioned into gene-rich euchromatic arms and constitutive heterochromatin [108]. Therefore, the structural complexity of chromatin, caused by inter- and intrachromosomal interactions identified as TADs, may increase the sensitivity to genotoxic stress, thereby acting as one of the factors determining DNA damage tolerance.

Individual chromosomes occupy their own space within the cell nucleus, shaping “chromosome territories”, which are larger in the scale of chromatin compartmentalization than TADs [109]. “Chromatin compartment boundaries”, which arise due to the territory formation, may be another structural element of chromatin influencing DNA damage sensitivity. In animals, chromosomal rearrangements, including dicentrics, translocations, and large deletions, are considered to be lethal after DNA damage. The incidence of these rearrangements is associated with chromosomal territories; for instance, translocation frequently occurs when two DSB ends are merged at chromatin compartment boundaries between chromosomes [110]. This prompts us to speculate that a smaller number of boundaries are formed in *Arabidopsis*, which lacks obvious TAD formation, thereby lowering its risk of suffering fatal translocations. This could be one explanation why *Arabidopsis* can survive higher doses of irradiation than animals. Future studies will precisely estimate the 3D chromatin architecture in plants and animals and identify the cause of DNA damage resistance of plant cells. Additionally, it will be important to explore plant-specific mechanisms to organize higher-order chromosomal architecture, which hormonal signals may control, to illuminate unique strategies of coping with genotoxic stress [111,112,113].

Originally, “epigenetics” was a notion proposed from plant research, and various plant-specific epigenetic regulators have been identified so far [30,114]. Nevertheless, it remains largely unknown how and to what extent plants differ from animals in terms of epigenetic regulation of DDRs. The major obstacle has been technical difficulties in determining the chromatin state in plant cells. However, recent advanced techniques, such as high-throughput chromosome conformation method (Hi-C) and modification-specific intracellular antibodies (mintbodies), allow for the high-resolution detection of the chromatin structure and chromatin modifications in plants as well as in animals [108,115,116]. Further studies will identify the epigenetic factors involved in genome maintenance and local DNA repair/recombination processes through their interaction with repair machinery and cell-cycle regulators, thereby providing a new perspective on DDR mechanisms.

## Data Availability

Not applicable.
